# Improving Working Conditions to Promote Worker Safety, Health, and Wellbeing for Low-Wage Workers: The Workplace Organizational Health Study

**DOI:** 10.3390/ijerph16081449

**Published:** 2019-04-24

**Authors:** Glorian Sorensen, Susan Peters, Karina Nielsen, Eve Nagler, Melissa Karapanos, Lorraine Wallace, Lisa Burke, Jack T. Dennerlein, Gregory R. Wagner

**Affiliations:** 1 Department of Social and Behavioral Sciences, Harvard T.H. Chan School of Public Health, Boston, MA 02115, USA; sepeters@hsph.harvard.edu (S.P.); Eve_Nagler@dfci.harvard.edu (E.N.); 2 Dana-Farber Cancer Institute, Boston, MA 02215, USA; Melissa_Karapanos@dfci.harvard.edu (M.K.); Lorraine_Wallace@dfci.harvard.edu (L.W.); Lisa_Burke@dfci.harvard.edu (L.B.); 3 Institute for Work Psychology, Sheffield University Management School, University of Sheffield, Sheffield S10 2TN, UK; k.m.nielsen@sheffield.ac.uk; 4 Bouvé College of Health Sciences, Northeastern University, Boston, MA 02115, USA; j.dennerlein@northeastern.edu; 5 Department of Environmental Health, Harvard T.H. Chan School of Public Health, Boston, MA 02115, USA; gwagner@hsph.harvard.edu

**Keywords:** occupational safety and health, prevention and protection, psychological wellbeing, safety culture, job stress, musculoskeletal disorders

## Abstract

This paper addresses a significant gap in the literature by describing a study that tests the feasibility and efficacy of an organizational intervention to improve working conditions, safety, and wellbeing for low-wage food service workers. The Workplace Organizational Health Study tests the hypothesis that an intervention targeting the work organization and environment will result in improvements in workers’ musculoskeletal disorders and wellbeing. This ongoing study is being conducted in collaboration with a large food service company. Formative evaluation was used to prioritize outcomes, assess working conditions, and define essential intervention elements. The theory-driven intervention is being evaluated in a proof-of-concept trial, conducted to demonstrate feasibility and potential efficacy using a cluster randomized design. Ten worksites were randomly assigned to intervention or control conditions. The 13-month intervention uses a comprehensive systems approach to improve workplace policies and practices. Using principles of participatory engagement, the intervention targets safety and ergonomics; work intensity; and job enrichment. The evaluation will provide a preliminary assessment of estimates of the intervention effect on targeted outcomes and inform understanding of the intervention implementation across worksites. This study is expected to provide insights on methods to improve working conditions in support of the safety and wellbeing of low-wage workers.

## 1. Introduction

Low-wage workers are often employed in jobs that pose significant health and safety risks [1,2,3]. Defined as work that earns two-thirds or less of the national median gross hourly earnings [4], the prevalence of low-wage work varies by country and is on the rise, notably in Germany, the Netherlands, the United Kingdom, and the U.S. [5,6]. Women, young workers, those with low levels of education, and immigrants are overrepresented in these jobs [4,7,8,9,10]. These positions are often characterized by job insecurity; uncertainty around work hours, contributing to instability in earnings; significant physical work demands; repetitive work; and low job decision latitude and autonomy [11,12,13,14]. Many low-wage workers are employed by contracting agencies or firms that then contract out specific services to “host” employers, thereby creating multiple layers of responsibility for workplace policies and working conditions [15]. These workers, also referred to as distributed workers because they work away from their organization’s central work location at least some of the time [16], may face additional obstacles in accessing company resources and support.

Studies of interventions to improve the working lives of low-wage workers in the U.S. are rare. The study described here was conducted in the U.S. food service industry, which employs 9.5 million workers [17], many of whom work in low-wage jobs with limited access to company resources and support. These workers include a large number of immigrants; approximately 22% of Hispanic immigrants in the U.S. are employed in food service and service-related occupations. In addition, only 1.8% of workers in the food industry are represented by a labor union [5]. A recent study found that among U.S. food preparation and service workers, fewer than one-quarter can use paid sick days due to job tenure requirements [18].

Organizational interventions are receiving increasing attention as a potentially sustainable and effective approach to improving worker safety, health, and wellbeing. Recent studies and reviews have suggested promising results for these organizational interventions [19,20,21,22,23,24], including for low-wage workers [11,25,26]. These interventions have focused on providing workers with greater decision latitude and autonomy, and on enhancing teams and leadership, and have demonstrated improved wellbeing [26,27,28], psychological health [26,29,30,31,32], and reduced sickness absence [29,32].

This paper describes an organizational intervention designed to improve the health, safety, and wellbeing of food service workers, and has implications for the health and wellbeing of low-wage workers in other industries. This intervention employed participatory strategies to engage multiple levels of management as well as employees as active agents in the change process [24,33]. Using this participatory approach, workers and their managers collectively gain resources, knowledge, and skills to identify workplace problems, develop solutions, and implement changes to improve their working conditions. This approach has also been found to increase job control and decision latitude [28,29,34], and enhance team functioning, communication, and leadership [26,35,36].

The objective of this paper is to describe the background, study design, intervention approach, and evaluation methods for the Workplace Organizational Health Study. This study was designed to address a significant gap in the literature regarding the impact of organizational improvements in policies and practices for the health, safety, and wellbeing of low-wage workers. By using a rigorous research design to test a theory-driven model that is further informed by practice and a close collaboration with a large food service company, we expect this study to shape future interventions for low-wage workers by providing a model for reducing disparities in worker safety, health, and wellbeing.

## 2. Materials and Methods

### 2.1. Study Design Overview

The Workplace Organizational Health Study was designed to test our a priori central hypothesis that an intervention targeting the work organization and environment, as well as individual safety practices and behaviors, would show promising improvements in the study’s primary outcomes: musculoskeletal disorders (MSDs), including pain and injury; and worker wellbeing, operationalized to include overall wellbeing, work-related wellbeing, and life satisfaction. The study’s specific aims were to: (1) identify working conditions expected to be associated with these study outcomes and which could be feasibly modified through changes in management practices; (2) determine the feasibility and potential efficacy of an organizational intervention designed to improve working conditions, and workers’ MSD symptoms and wellbeing; and (3) understand variations in intervention implementation that in turn influence the outcomes. Baseline data were collected in July–August 2018, and the intervention was launched in October 2018; post-intervention data will be collected November–December 2019.

**Figure 1:**
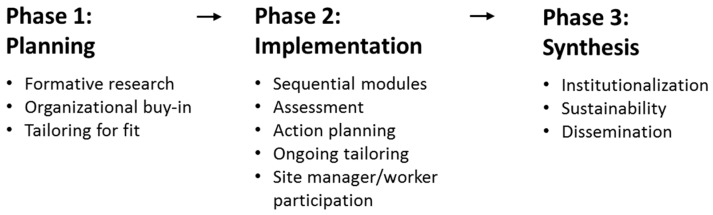
Intervention Framework.

Figure 1 illustrates the intervention framework and sequence, described in detail below. Because organizational policies and practices are managed across different levels of the organization, we engaged stakeholders at the national, district, and worksite levels within this large company. During the Planning Phase, we collaborated with company representatives at the national and district levels to ensure their support, leverage resources at the national and district levels, and facilitate buy-in from participating worksites. We conducted formative qualitative research to prioritize outcomes, assess working conditions, and formulate essential intervention elements. In the Implementation Phase, we evaluated the worksite-based intervention in a proof-of-concept (PoC) trial. A PoC trial is conducted to demonstrate feasibility or potential efficacy, typically on a small scale as a milestone toward full development of a “concept” [37,38,39,40]. Using a cluster randomized design, 10 worksites were randomly assigned to intervention or control conditions. The worksite-based intervention focused on improving three working conditions: safety and ergonomics; work intensity; and job enrichment. At the time of writing this paper, this study was implementing the intervention. During the Synthesis Phase, the research team and collaborating company will review findings and develop strategies to institutionalize, sustain, and disseminate successful intervention components. Changes related to these intervention processes will be evaluated following methods described in the Evaluation section.

This study obtained ethics approval through the Harvard T.H. Chan School of Public Health Institutional Review Board (Protocol #IRB16-0488). Participants provided informed consent prior to participating in any data collection.

### 2.2. Study Population and Sample

This study took place in a large multinational company that provides food service through contractual arrangements with corporate clients. The food service worksites in this study are located in corporate settings in or near Boston, Massachusetts (U.S.). These worksites are organized by district, based on geographical location.

**Figure 2:**
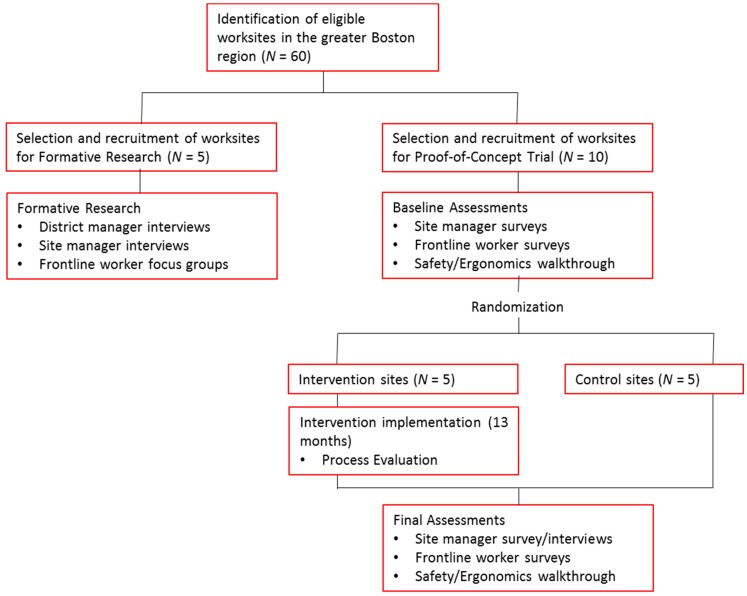
Study design, settings, and samples.

The study design, setting, and sample are illustrated in Figure 2. For both the formative research and PoC trial, eligible worksites employed between 7 and 30 employees. Worksites participating in the formative research were not eligible for the PoC study, to avoid potential contamination arising from exposures to the topics raised in the qualitative research. Worksites eligible to participate in the PoC trial were required to have a contract that was expected to last through the intervention period; agree to the planned data collection efforts; and agree to be randomly assigned to the intervention or control condition following the baseline survey.

Frontline workers included chefs, cooks, food preparers, servers, dishwashers, and cashiers. Frontline workers in five non-PoC worksites participated in the formative research. In the PoC trial, frontline workers participated in surveys and the intervention. At baseline, approximately half of the participating workforce was female, and the average age of the frontline workers was 43.6 ± 13.07 years (range, 20–72 years). This multi-ethnic workforce included approximately one-third who identified as Hispanic; 16% as Black or African American; 9% as Native American, Asian, Native Hawaiian, or Pacific Islander; and 45% as White.

Two groups of managers were also included. Site managers were responsible for operations at each worksite. Site managers at the formative research worksites were interviewed as part of the planning process. For the PoC trial, site managers at all 10 PoC worksites participated in the site manager survey and were interviewed as part of the safety and ergonomics walkthrough. The five site managers at PoC intervention worksites were central to all phases of the intervention planning and implementation. District management included those with responsibilities across worksites at the district level, including representatives of human resources, health and safety, operations, and those with direct supervision of the participating worksite accounts. District managers were interviewed as part of the formative research, and for the intervention, contributed to planning and implementation of district-level policies and practices.

### 2.3. Phase 1: Planning

This study was conceptualized jointly by the research team and the collaborating company, with company leadership provided by a national representative who facilitated dialogue with key company stakeholders at the national and district levels. Guided by a conceptual model and formative research, we identified the priorities and needs of frontline workers and the worksites that employed them to inform intervention development.

#### 2.3.1. Formative Research

We used formative research [41] to inform priorities for our research outcomes and intervention methods, including identifying working conditions to be targeted by the intervention [42]. Conducted in Year 1 of the study (2017), we used qualitative methods to address four key questions [42]: (1) What do frontline workers and managers employed by a food service company perceive as priorities for their health, safety, and wellbeing (outcomes of interest)? (2) What are the potential root causes (working conditions) of the health, safety, and wellbeing concerns identified? (3) What mechanisms are likely to facilitate intervention processes and success (intervention working mechanisms)? (4) What does management consider feasible targets of the intervention, including changes in organizational policies, programs, and practices (prioritization)? The formative research also contributed to understanding how cognitive and motivational biases may influence the intervention, allowing us to plan accordingly [43].

The formative research included semi-structured interviews with district and site managers, focus groups with frontline workers using an interview/focus group guide, and observations of the worksites. Findings (published elsewhere [42]) were synthesized to inform the intervention design in collaboration with representatives of the employer as part of an intervention planning and prioritization workshop.

#### 2.3.2. Conceptual Model

To inform intervention development and research planning, we incorporated information from our formative research and an overall conceptual model developed previously by the research team [44]. This model relies on several theoretical perspectives, including the social ecological model [45,46], social contextual model of health behavior change [47,48], hierarchy of controls [49,50,51], and participatory frameworks [52]. Our evaluation approach was based on both the RE-AIM [53] and realist evaluation theoretical frameworks [54]. These theoretical foundations underscore the complex interplay of workers, their work environment, as well as characteristics of the larger contexts in which both the worker and the worksite are embedded. This overall model places working conditions, including physical, organizational, and psychosocial factors at work, as central determinants of health and safety outcomes as well as enterprise/organizational outcomes. Working conditions function as a pathway from policies, programs, and practices to worker and enterprise outcomes.

**Figure 3:**
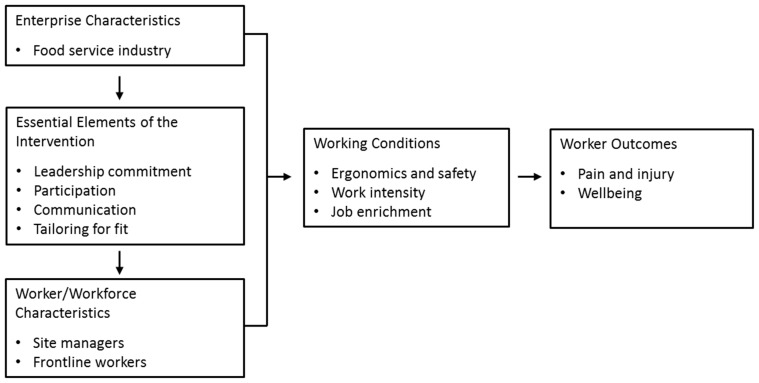
Conceptual Model.

Maintaining this central focus on working conditions, in this study we operationalized the overall model to focus on this intervention and setting (Figure 3). The model illustrates the essential intervention elements, working conditions, and outcomes specifically targeted within this intervention. Through formative research, workers and managers identified core priorities for their safety, health, and wellbeing. Following the pathways in the conceptual model, we identified working conditions likely to influence these outcomes, and intervention elements essential to improving these working conditions.

Three working conditions were identified to be targeted by this intervention. Safety and ergonomics included risks associated with slips, trips, and falls and housekeeping; job requirements that included reaching, lifting, pushing, pulling, and carrying demands; sedentary behaviors such as standing for long periods; and equipment use. Work intensity encompassed work flow, workload, and pace of work; organization of job tasks and demands; and decision latitude and decision making associated with completion of job tasks. Job enrichment addressed needs for role clarity and setting clear expectations; opportunities for professional development and career advancement; and supportive practices associated with providing feedback on job performance.

Previous research has confirmed the important contributions of the work organization to worker safety, health, and wellbeing [2,11,19,21,24,55]. We relied on four underlying essential elements in the organizational change process [56]. Through leadership commitment, the aim was to provide necessary resources and support, ensure accountability, and establish health, safety, and wellbeing as an organizational priority. Leadership commitment has been associated with improved outcomes related to job-related wellbeing [57,58], workplace injuries [59,60], and health behaviors [61,62]. Participation includes engaging stakeholders at every level of the organization, including district-level managers, site managers of specific worksites, and frontline workers. This combined approach of using both top-down and bottom-up approaches in the intervention development and implementation builds on assets and resources across multiple levels of the organization [33,63,64,65]. Communication provides a vehicle for building collaborative relationships across the organization, including fostering effective vertical and horizontal communications [33,66]. Tailoring for fit reflects the need to tailor the intervention to the organizational context [56]. Integrating interventions into existing structures and practices may minimize additional burdens arising from implementing new procedures and from changes in the ways work is organized, designed, and managed [56].

#### 2.3.3. Organizational Buy-In and Fit

Throughout the study, the research team met regularly with national representatives of the collaborating company, and engaged district-level leadership in planning the intervention, accessing company resources, and providing feedback to guide decisions about policies and practices. Leadership sets the overall vision for the organization, engages management support at multiple levels, and ensures that necessary resources are available to support targeted changes [55]. In this study, key stakeholders contributed to the design of the intervention, ensured fit with organizational priorities, linked the research team with central resources that are not uniformly accessed in the worksites, and contributed to problem solving and addressing barriers and challenges to intervention implementation. A coordinating committee was established to engage stakeholders at the district and national levels in reviewing relevant policies and practices, ensuring ready access to necessary resources, and coordinating efforts with site managers across the five intervention worksites.

### 2.4. Phase 2: Intervention Implementation

The intervention model applied the implementation guidelines developed by the Harvard T.H. Chan School of Public Health Center for Work, Health, and Well-being [67]; findings from a scoping review of the literature on prior organizational interventions in low-wage service settings [68]; and systematic formative research, described above and elsewhere [42]. The 13-month intervention used a comprehensive systems approach to improve worker health by targeting workplace policies and practices, focusing here on the essential intervention elements identified in the formative research as critical drivers of the workers’ health, safety, and wellbeing outcomes.

To support the implementation of the intervention, a member of the research team met at least monthly with the site managers of each of the five participating worksites. The intervention was organized around three sequential modules defined by the targeted working conditions: safety and ergonomics, work intensity, and job enrichment. Worksite-specific assessments informed the intervention process. For the safety and ergonomics module, the safety and ergonomics walkthrough assessment identified site-specific hazards and made recommendations to guide priority setting. For the work intensity and job enrichment modules, a standard set of questions guided interviews with site managers to identify current practices, potential challenges, and opportunities and resources. The research team developed a report based on each assessment, which was used in priority setting and as the foundation for action planning. We followed a standard protocol for the intervention process for the three modules, including meetings and regular communications with the site manager to conduct the assessment, identify priority actions needed to address recommendations from the assessment, engage frontline workers, and outline and implement a work plan to determine necessary tactics and timelines.

Following principles of participatory engagement [69,70], the research team worked with site managers and frontline workers at intervention sites to tailor the intervention at each location. The participatory process included collaboration with leadership at all levels; regular meetings with site managers of the individual sites; and communications and brief meetings with frontline workers. The participatory process provided a way to facilitate an open and constructive interaction between managers and employees about the main concerns in their worksite, thereby informing priorities for the intervention. The expectation was that with increasing engagement, communications would be strengthened and the organization itself would have increasing capacity to implement organizational changes. In addition, we used participatory processes for tailoring this intervention to this population of low-wage workers. For example, we developed tools for designing and implementing action plans that considered the time pressures faced by these workers. We also provided guidance and technical assistance to managers on how to facilitate a participatory process, focusing specifically on ways to engage this population of low-wage workers.

### 2.5. Phase 3: Synthesis

This study is expected to increase understanding of how to improve workplace policies and practices to support the safety and health of low-wage workers effectively and sustainably [71]. The research team will work with the collaborating company to understand the research findings and lessons learned, identify successes and ongoing challenges, and incorporate organizational changes relevant to the work lives of low-wage workers, as part of system-wide changes. Together, we will identify opportunities for institutionalizing the intervention approach and lessons learned into sustainable organizational policies, programs, and practices. This collaboration with a leading employer of low-wage workers promises to provide a generalizable approach for dissemination of lessons learned both within the company and across this industry sector, with significant implications for other low-wage settings.

### 2.6. Evaluation of the Intervention

The evaluation will: (1) provide a preliminary assessment of estimates of the effect of the intervention on targeted outcomes, and (2) inform understanding of the intervention implementation across worksites and identify possible contextual factors that may explain variations [24,72,73].

*Intervention effectiveness assessed in the PoC study—Does it work?* We used the cluster randomized controlled trial design to achieve the first aim. As illustrated in Figure 2, following completion of a baseline survey, worksites were blocked on size (fewer than 15 employees versus 15 or more employees), and randomly assigned to intervention or control conditions, with five sites per group. We will evaluate changes in primary outcomes between the baseline and final surveys and compare observed changes between intervention and control sites. Baseline surveys were conducted with site managers and frontline workers using well-established measures from our prior research and the broader literature; these procedures will be followed again post-intervention. All data were collected by trained evaluation staff who are independent from intervention delivery.

All frontline workers were eligible to participate in the worker survey. This 30-minute interviewer-administered survey was conducted on-site during work time in English or Spanish. The baseline survey included 119 workers (response rate = 91.5%). Wellbeing was operationalized as general wellbeing [74], including overall life satisfaction [74,75]; work-related wellbeing, adapted from a measure of flourishing [76]; and job satisfaction, measured using a standard single item measure [77]. MSD symptoms were measured using standard validated measures of pain [78], reported injuries [79], and functional limitations [80]. We assessed two secondary outcomes: turnover intention [81] and employee engagement [82]. We also measured workers’ self-reports of working conditions, including supervisor support [83], involvement of employees [83], decision-making latitude at work [83], possibilities for career development, safety practices [84], quantitative job demands [85], physical activity at work [86], and ergonomic practices [84]. The survey also assessed job characteristics and demographics.

Site managers were surveyed at baseline and follow-up in all 10 PoC sites to evaluate changes in implementation of policies and practices. This survey included the Workplace Integrated Safety and Health Assessment [55], which measures six key organizational characteristics contributing to intervention success that are well-aligned with the study’s essential intervention elements: leadership commitment; participation; policies, programs, and practices fostering worker safety, health, and wellbeing; comprehensive and collaborative strategies; adherence; and data-driven change. Additional measures included measures in parallel with the worker survey.

We also conducted a safety and ergonomics assessment in the ten PoC worksites at baseline and follow-up using a qualitative tool to identify specific risks in the physical work environment, including housekeeping and other factors that contribute to slips, trips, and falls; and physical work demands such as lifting, bending, or stooping, awkward postures, standing for long periods, and repetitive or forceful tasks [87]. The pre-intervention assessment in intervention sites was used to inform an action planning process as part of the intervention.

We will estimate the changes in the outcomes between baseline and follow-up surveys using mixed effect linear modeling methods with time (baseline or follow-up) and intervention condition (intervention or control). In addition, we will further compare changes in the work organization and environment using data from the walkthrough assessments, manager interviews, and frontline worker surveys.

*Understanding variations in intervention implementation—What works for whom in which circumstances?* To complement assessments of intervention effectiveness, we will use principles of realist evaluation to design an evaluation of what works for whom in which circumstances [54]. The evaluation aims to identify contextual factors that are likely to trigger the intervention’s mechanisms to bring about the intended outcomes. Contextual factors include existing policies, practices, and relationships, as well as events, such as changes in leadership, which may facilitate or hinder the change process [26]. These factors will be examined in relation to outcomes, collected through the baseline and follow-up surveys as described above and supplemented with a parallel mixed methods data collection approach [88]. As illustrated in Figure 3, we will track implementation of the essential elements of the intervention (leadership commitment, participation, communication, and tailoring for fit), as well as intervention content focusing on the working conditions targeted by the intervention (safety and ergonomics, work intensity, and job enrichment) [54]. By assessing implementation of these components, we will be able to assess the feasibility of the intervention, including the extent to which interventions are delivered as planned and in which dose, following principles of process evaluation [89]. Data are being collected from the five intervention worksites at multiple levels, at multiple time points, and through different modalities using a mix of qualitative and quantitative methodologies, thereby enabling us to triangulate data and understand variations in intervention implementation.

The process evaluation includes quantitative and qualitative data. Quantitative data include checklists tracking meetings with site managers and the district-level coordinating committee and documenting key intervention activities. Qualitative meeting minutes from these and other intervention-related meetings and communications provide information on the wider context of the intervention. Tracking the extent to which action plans are being implemented as planned and tailored to the worksite context enable us to assess mechanisms in the process of change. We also monitor participation in brief meetings with workers and site managers and explore whether engagement in these meetings enables participation and supports implementation of action plans. Members of the research team observe meetings and record reflections on process mechanisms, such as the extent to which workers are able to influence the intervention through participatory processes. Qualitative data also include interviews with key stakeholders, including interviews with national and district representatives conducted during and at the end of the intervention, to explore the impact of contextual factors on the implementation of the intervention [90,91,92]. In parallel, we will conduct focus groups with workers in the five intervention sites using an open-ended moderator guide to explore their perceptions of the acceptability and feasibility of the intervention process.

All qualitative data, such as audio-taped and transcribed individual interviews and focus groups, and meeting data collection materials, will be analyzed using the software program *NVivo*(QSR International Pty Ltd. Melbourne, Australia). [93]. Data will be coded using template analysis [94], including using themes based on the intervention content (i.e., working conditions—safety and ergonomics, work intensity and job enrichment), process mechanisms (i.e., essential intervention elements—leadership commitment, participation, communication, and tailoring for fit), and contextual factors. Quantitative process tracking data will be analyzed using SPSS (IBM, Armonk, NY, USA) to allow for integrative analyses with the site manager and worker surveys [93,94]. Assessment of the relationships among the intervention content, context, mechanisms, and outcomes will be tested using mixed methods analysis [95] and integrative statistical analyses (e.g., multi-group structural equation modeling) [96].

## 3. Discussion

Low-wage workers often encounter both organizational and physical risks in their work environments. The Workplace Organizational Health Study is expected to contribute to improved understanding of the intervention mechanisms, feasibility, and potential benefits of modifications in the work organization that may contribute to improvements in safety and wellbeing for low-wage workers. This study was guided by a conceptual model that identifies working conditions as pathways to protecting and promoting worker health [44]. This innovative focus on improvements in working conditions holds significant promise for employers and their employees alike.

This mixed methods study used formative research to inform intervention priorities and approaches. We collected qualitative and quantitative data from managers and workers to provide a broad-based perspective of factors in the work organization that were targeted by this intervention [42]. Managers, at both the site and district levels, are central to making improvements in the work organization, and significantly influence the implementation of policies and practices [97]. Furthermore, workers’ perspectives of the work organization, including policies, how they are implemented, and the working conditions of most relevance to them, are central to planning effective interventions.

This study explores the feasibility and potential efficacy of the intervention through a PoC trial as a step in the process of intervention development and testing. Preliminary testing in the relatively small scale of a PoC trial provides important advantages, although ultimately, it will be important to demonstrate efficacy and effectiveness in a full-scale cluster randomized controlled trial (RCT), adequately powered across multiple worksites. Launching a full-scale RCT requires sufficient preliminary data to document potential efficacy of the intervention, data provided through this design. The randomized controlled design used in the PoC will provide estimates of the size of intervention effect, allow us to isolate the effect of the intervention on observed changes relative to the control group, and provide information on the feasibility of these organizational changes. This study also benefits from the application of principles of realist evaluation [54,98]; within the intervention group, we will explore what works for whom in which circumstances. As illustrated in our conceptual model, we will explore the process mechanisms influencing these outcomes, including the role of essential intervention elements (leadership commitment, participation, communication, and tailoring for fit), and identify the extent to which working conditions (ergonomics and safety, job enrichment, and work intensity) improve as a result of these changes.

This study is based on a collaboration with a large employer with significant global reach and a willingness to support changes in the work organization as part of this collaboration. This has meant that we have had strong leadership commitment in the intervention development phases. Although implementing this study with a single employer limits generalizability, this collaboration provides an opportunity for designing and testing this organizational intervention in an employment setting representative of that experienced by many low-wage workers (e.g., type of work common in the service sector, low unionization rates, contracting employer) [5]. In this early stage research, we believe it is appropriate to restrict our sample to this single setting in order to minimize extraneous factors contributing to observed outcomes while also leveraging the potential impact of strong partnership engagement. In addition, these results will have potential applicability to the large number of food service workers employed by this company across multiple countries, and to other work settings that employ low-wage workers that share similar characteristics with regard to organizational policies supportive of worker health outcomes.

The distributed nature of this work setting, based on contractual relationships between the collaborating company and contracting clients where the food services are operated, provides a view into the “fissured workplace” [15]. This setting illustrates the complex nature of these relationships [99,100]. As part of a large multi-national organization, site managers must balance the demands of the parent organization and the local demands of their clients. In addition, these worksites face significant competition for maintaining the contracts with their client, contributing to ongoing pressures to deliver optimal service at minimal cost. The work pace and hours of operation set by the client also may contribute to work intensity. These multiple sources of stress contribute to the overall working climate, not only for low-wage workers but also across all hierarchical levels [101]. The contract between the service-providing organization and the client likely specifies who is responsible for compliance with occupational health and safety laws as well as food safety and other regulations. Assuring compliance with these laws and regulations is the joint responsibility of the employer and the organization contracting for their services [102]; nonetheless, ensuring adequate worker protections can be challenging in the context of such “non-traditional” employment relationships.

## 4. Conclusions

This paper describes the study design, intervention process, and evaluation plans for the Workplace Organizational Health Study, which includes a proof-of-concept trial to test the feasibility and potential efficacy of organizational approaches to improve the health, safety, and wellbeing of low-wage frontline food service workers. This research also systematically explores mechanisms influencing intervention implementation in order to understand what works for whom in which circumstances [54]. This study is expected to provide innovative insights into methods to improve working conditions in support of the safety, health, and wellbeing of low-wage workers. The collaboration described here may allow us to identify strategies for maximizing fit between the intervention and the organization in order to incorporate changes in working conditions relevant to the lives of low-wage workers. Improving working conditions for low-wage workers may help to create an environment where workers can thrive, where safety practices are facilitated, and where health and safety behaviors are supported by a culture of health [19,44].
